# Low Vitamin D Level Is Associated with Acute Deep Venous Thrombosis in Patients with Traumatic Brain Injury

**DOI:** 10.3390/brainsci11070849

**Published:** 2021-06-25

**Authors:** Matthew Moore, Yelena Goldin, Harsh Patel, Brian D. Greenwald

**Affiliations:** 1 Department of Physical Medicine and Rehabilitation, JFK Johnson Rehabilitation Institute, Edison, NJ 08820, USA; yelena.goldin@hmhn.org (Y.G.); Brian.Greenwald@HMHN.org (B.D.G.); 2 Rutgers Robert Wood Johnson Medical School, New Brunswick, NJ 08901, USA; h.h.patel19@gmail.com

**Keywords:** deep venous thrombosis, traumatic brain injury, vitamin D

## Abstract

Vitamin D and its association with venous thromboembolism (VTE) have been studied in common rehabilitation populations, such as spinal cord injury and ischemic stroke groups. This study explores the relationship between vitamin D levels and acute deep venous thrombosis (DVT) in the traumatic brain injury (TBI) population. This is a retrospective cohort study that analyzes the relationship between vitamin D levels and the prevalence of DVT during acute inpatient rehabilitation. In this population, 62% (117/190) of patients had low vitamin D levels upon admission to acute rehabilitation. Furthermore, 21% (24/117) of patients in the low vitamin D group had acute DVT during admission to acute rehabilitation. In contrast, only 8% (6/73) of patients in the normal vitamin D group had acute DVT during admission to acute rehabilitation. Fisher’s exact tests revealed significant differences between individuals with low and normal vitamin D levels (*p* = 0.025). In conclusion, a vitamin D level below 30 ng/mL was associated with increased probability of the occurrence of acute DVT in individuals with moderate–severe TBI.

## 1. Introduction

Venous thromboembolism (VTE), presenting in the form of deep venous thrombosis (DVT) and pulmonary embolism (PE), has shown increased prevalence in traumatic brain injury (TBI) populations [1]. Virchow’s triad (stasis, hypercoagulable state, and endothelial injury) is common in trauma patients presenting to the emergency department with TBI [1]. In severe trauma, bleeding causes the release of coagulation factors that can lead to VTE [1]. Physical and cognitive deficits in combination elevate the risks of developing VTE. TBI patients are often immobile, weak, and bedbound, and suffer from other traumatic injuries that increase the risk of venous stasis. A 2009 study found that there is a three-to fourfold increased risk of DVT in TBI patients [2]. Well’s score and number of days in bed were shown to be significant predictors for developing DVT in neurosurgical patients [3]. Another study showed that major general surgery and major trauma were strong risk factors for the development of VTE, while prolonged bed rest was a weak risk factor [4].

Initiation of chemical DVT prophylaxis may be delayed in TBI patients secondary to the risk of worsening intracranial hemorrhage. Delaying prophylaxis can increase the risk of morbidity and mortality in the form of VTE. The current literature states that chemoprophylaxis is not appropriate for patients with spontaneous ICH expansion during the first 72 h post injury [5]. Despite the increased prevalence of VTE in this patient population, a universally accepted standard for chemoprophylaxis management does not exist. Initiating low-molecular-weight heparin or unfractionated heparin is often a decision made by the neurosurgery or trauma team in an acute care hospital [6]. Bradley et al. studied the effects of initiating chemical DVT prophylaxis in TBI patients with intraparenchymal pressure monitors in place. They found no new hemorrhages or hemorrhage expansion with low-molecular-weight heparin or unfractionated heparin [7].

Vitamin D is an essential nutrient that helps maintain many important bodily functions. Calcium homeostasis, skeletal integrity, and neurodevelopment all benefit from adequate vitamin D levels [8]. In the general population, high vitamin D levels have been shown to be associated with younger age, better nutrition, and increased physical activity [9,10,11]. Vitamin D deficiency has been linked to problems such as dementia, depression, diabetes mellitus, autism, and schizophrenia [8]. When exploring optimum vitamin D levels for health, most studies focus on fracture prevention and the preservation of bone mineral density. A consensus from these studies states that serum 25-hydroxy vitamin D (VitD-25OH) concentrations should exceed 75 nmol/L (30 ng/mL) [12]. Current nutrition guidelines in addition to sun avoidance for skin cancer prevention make it difficult for adults to maintain adequate levels of vitamin D without supplementation [12].

Numerous studies have established a pattern of vitamin D deficiency in TBI populations. Pellicane et al. published two studies in 2010 and 2011 that showed increased prevalence of VitD-25OH deficiency in rehab populations and TBI patients in particular. The 2010 study showed that 66% of outpatient rehabilitation patients were VitD-25OH deficient [13]. Lower vitamin D levels were associated with non-white race, history of spinal cord injury, TBI, and hereditary musculoskeletal diagnosis [13]. A similar study in 2011 showed that 77% of admissions to acute rehabilitation were VitD-25OH insufficient or deficient [14]. Intiso et al. established that disability severity was correlated with vitamin D deficiency in severe TBI [15]. Poor nutrition and decreased sun exposure may contribute to decreased VitD-25OH levels. However, there may be a secondary phenomenon occurring resulting from the inflammatory processes of TBI [15].

The relationship between vitamin D levels and the development of VTE has been studied. A 2014 case–control study of 82 participants showed that low levels of VitD-25OH were associated with idiopathic lower extremity DVT [16]. A 2019 cross-sectional study evaluated vitamin D levels in 42 patients with lower extremity DVT or PE vs. 42 controls. They concluded that patients with VTE had lower concentrations of vitamin D [17]. Prior studies have explored the relationship between low vitamin D levels and VTE in other populations typically found in acute rehabilitation. A 2019 study investigated vitamin D deficiency, supplementation, and the development of VTE in spinal cord injury patients. This study showed that, while there was no significant difference in the development of VTE in VitD-25OH deficient patients, those supplemented with vitamin D were less likely to have VTE [18]. Wu et al. published a study in 2018 that showed that vitamin D deficiency was independently associated with the development of DVT in ischemic stroke [19]. The purpose of this study was to examine the relationship between vitamin D levels and the development of DVT in acute rehabilitation patients with TBI.

## 2. Materials and Methods

### 2.1. Study Population

This is a retrospective cohort study of patients admitted into acute inpatient rehabilitation. Patients were admitted between December 2016 and December 2018. A total of 200 patients with the diagnosis of TBI were included in the study database. Individuals were managed in a specialized rehabilitation unit focusing on TBI patients. All new admissions to acute rehabilitation are routinely screened for low vitamin D levels and lower extremity DVT. This is a departmental policy that was established due to the high prevalence of both conditions. Demographic data, medications, vitamin D level, and bilateral lower extremity venous Doppler results were retrospectively extracted from the electronic medical record. The study recruited 190 individuals with moderate or severe traumatic brain injury based on continuous admissions for acute inpatient unit between December 2016 and December 2018. TBI severity was determined based on the duration of loss of consciousness, GCS scores, and the duration of post-traumatic amnesia. Ten patients were excluded from study because of incomplete data.

### 2.2. Vitamin D Levels

Serum 25-hydroxy vitamin D levels were collected upon admission for all patients. Blood samples were drawn and collected in a 3.5 mL gold-top serum separator tube and transported to the lab. Results were obtained by electrochemiluminescence immunoassay (ECLIA) using a cobas 6000 from Roche Diagnostics (Basel, Switzerland). Data were transferred to the EMR via the laboratory technician. If a patient’s VitD-25OH level was not checked on admission, they were excluded from the study. Most experts agree that vitamin D deficiency is defined as having a VitD-25OH concentration less than 20 ng/mL and vitamin D insufficiency as VitD-25OH concentration between 21 and 29 ng/mL [20]. These guidelines were formulated by a collection of studies that focused on minimizing falls and fractures.

### 2.3. DVT Status

Upon admission to acute rehabilitation, patients underwent a bilateral lower extremity venous Doppler to screen for DVT. Presence of acute DVT in the thigh or calf was accepted as significant for the purpose of the study. If an upper extremity venous Doppler was performed during their admission, any acute DVT was accepted as significant. Chronic DVT or superficial venous thromboembolism were not accepted as significant for the purpose of this study. Patients often received multiple venous Doppler studies of their extremities for various reasons. All subsequent studies were included in the data collection process. If a patient did not have a lower extremity venous Doppler during their stay, they were excluded from the study.

### 2.4. Statistical Analysis

One-way analysis of variance (ANOVA) was used to examine the differences in age, education, and length of stay between the vitamin D status groups. Fisher’s exact test (two-tailed) was used to determine sex differences between groups. Two-tailed Fisher’s exact tests were used to examine the relationships between (1) vitamin D status and DVT, (2) vitamin D status and vitamin D supplementation, and (3) vitamin D supplementation and DVT within each vitamin D group. *p*-values of 0.05 or lower were considered statistically significant.

## 3. Results

Demographic variables and length of stay data for the entire sample are tabulated in Table 1 by vitamin D status. One-way ANOVAs (for continuous variables) and Fisher’s exact tests (for categorical variables) revealed that individuals with insufficient and deficient vitamin D levels were significantly younger than individuals with WNL levels (F = 4.2, *p* = 0.015). There was also a significant relationship between vitamin D level and length of stay in acute inpatient rehabilitation, with post-hoc tests revealing that this difference was attributable to significantly longer length of stay of individuals with vitamin D deficiency compared to that of individuals with WNL vitamin D (F = 4.1 *p* = 0.018). There were no significant differences in education (F = 1.1, *p* = 0.339) or sex (*p* = 0.517) between the vitamin D groups.

Vitamin D levels in individuals with TBI ranged from 5 to 98 (29 ± 14.9). Among the 62% of individuals with low (<30 ng/ML) vitamin D levels in our sample, one half (31% of the total sample) met criteria for insufficiency and the other half (31% of the total sample) met criteria for deficiency. The two groups were combined into one low vitamin D group. This is consistent with the approach used by Ehsanian et al. (2019) to evaluate the probability of VTE in individuals with spinal cord injury with low versus normal vitamin D levels. These results are presented in Table 2. Fisher’s exact tests revealed significant differences in the probability between individuals with low and WNL vitamin D levels (*p* = 0.025), suggesting that vitamin D levels below 30 ng/mL are associated with increased probability of DVT in individuals with acute moderate–severe TBI. 

Given significant differences in rehab LOS based on vitamin D status, a binary logistic regression was conducted to examine the relationship that LOS shares with the association between vitamin D groups (low vs. normal) and DVT. Rehab LOS was entered in Step 1 as a control variable, vitamin D group was entered in Step 2 as the primary predictor of interest, and DVT status was entered as a dependent variable. Results of the logistic regression revealed a significant association between rehab LOS and DVT (OR = 1.03, *p* = 0.01) and a non-significant association between the vitamin D group and DVT (OR = 2.39, *p* = 0.78).

## 4. Discussion

This study attempts to identify an association between acute DVT and vitamin D levels in TBI patients. When presented with the data, there is a statistically significant difference in the occurrence of acute DVT in patients with low vitamin D levels versus those with normal vitamin D levels. Why this relationship exists remains unclear, and resolving this question was not the purpose of this study. Vitamin D levels can serve as a measure of one’s health. Lack of consistent sun exposure can contribute to low vitamin D levels and may suggest a lack of outdoor activity and exercise. Many factors that contribute to normal vitamin D levels also make people less susceptible to DVT.

Brain trauma patients may suffer from dysphagia or altered mental status, which can limit nutritional intake. Severely injured patients with poor oral intake often have increased functional deficit. Early ambulation has been shown to prevent DVT in hospitalized patients [21]. One can see that nutritional status and degree of impairment are often related, where more severely injured patients require enteral nutrition and vitamin supplementation. Patients admitted to acute inpatient rehabilitation often need to have their diets and supplements adjusted in order to meet their goals. 

TBI patients often present to acute inpatient rehabilitation with additional injuries, with orthopedic injuries being among the most common. Studies have shown that fractures increase the risk of DVT [22]. Reasons for this include immobility, weight-bearing limitations, and hypercoagulable state. The combination of brain and orthopedic injury creates a scenario in which the risk for acute DVT dramatically increases. 

Vitamin D supplementation has been hypothesized to improve outcomes after TBI. Tang et al. studied the effects of progesterone and vitamin D combination therapy after TBI in rats. Combination supplementation showed greater efficacy in reducing neuroinflammation at 24 h after TBI [23]. Aminmansour et al. studied the effects of vitamin D and progesterone supplementation on patients with severe TBI. This was a randomized, placebo-controlled trial that showed that the recovery rate in patients receiving progesterone and vitamin D was significantly higher than in progesterone-only or placebo groups [24]. Lee et al. studied the relationship between vitamin D supplementation and clinical outcome in 345 mild-to-moderate TBI patients. They concluded that supplementation may improve long-term performance and cognitive outcomes [25]. A study by Jamall et al., which included 353 adult participants, concluded that vitamin D deficiency is common in patients after TBI and is associated with impaired cognitive function and depressive symptoms during the first outpatient visit following hospitalization [26]. 

The relationship between low vitamin D levels and increased prevalence of venous thromboembolism has been established and is mostly attributed to the reasons above. However, there are biochemical mechanisms that may contribute to this. Of most interest is the impact that vitamin D has on the coagulation cascade. Thrombomodulin, a cofactor for thrombin activation of protein c, inhibits the procoagulant effects of thrombin [27]. Tissue factor initiates the extrinsic pathway by activating thrombin [28]. Koyama et al. demonstrated that vitamin D upregulates thrombomodulin and downregulates tissue factor in some human cells [29]. Polymorphisms in the vitamin D receptor gene have been found to be statistically significant in the development of post-medical and post-surgical DVT [30]. This receptor is found in vascular endothelial cells, which further supports the role of vitamin D in anticoagulation [31]. Aihara et al. concluded that the vitamin D receptor was vital to maintaining homeostasis in mice models [31].

This study supports prior research that shows that low vitamin D levels and DVT are more prevalent in TBI patients. Within this population, it was found that those patients with low vitamin D levels on admission were more likely to have acute DVT than those with normal vitamin D levels. Vitamin D level was impacted by the length of stay in rehab. A secondary analysis showed there was a significant association between LOS in rehab and the development of DVT. Vitamin D levels and lower extremity Dopplers were collected within the first few days of admission, so it is not clear how valuable this analysis is in practice. One could investigate whether a longer LOS in rehab is associated with worse medical status or a longer length of stay in acute care, but such data were not available for analysis. Although it is difficult to determine causation, we must consider other factors as possible contributors to the development of DVT in future studies.

This study was not without limitations. Due to limitations of the electronic medical record, we were unable to determine if vitamin D supplementation was started before or after admission to acute inpatient rehabilitation. For this reason, data exploring the effects of vitamin D supplementation after admission to rehabilitation were not included in this study. Many other comorbidities also contribute to the development of DVT in hospitalized patients, such as lower extremity fractures, sacral ulcer, depression, sepsis, and neoplasm. Attempts were made to control for these conditions; however, due to limitations of the database and electronic medical record, this analysis was not included. The question of adequate DVT prophylaxis, such as the use of sequential compression devices (SCDs), low-molecular-weight heparin, and unfractionated heparin, can also be asked. Again, due to limitations of the electronic medical record and database, the timing and duration of prophylaxis could not be accurately determined. TBI severity could potentially play a role in the prevalence of DVT among this population. Although all patients in the database were considered to have moderate or severe TBI, our data were limited, and further analysis could not be completed in comparing severe TBI vs. moderate TBI in the development of DVT. This should be considered in future studies.

## 5. Conclusions

This retrospective cohort study supports the hypothesis that low vitamin D levels on admission to acute rehab are associated with acute DVT in TBI patients. There are many factors that may contribute to this finding, and further research must be carried out in order to determine why this relationship exists. Normal vitamin D levels have been established as an important marker of proper health and nutrition in prior and unrelated studies [12]. Vitamin D supplementation has been shown to improve outcomes in TBI patients in regard to quality of life and outcomes [24,25]. TBI patients are at high risk of medical complications both in the short term and long term. Maintaining proper nutrition through diet and supplementation is vital to achieving favorable outcomes after injury.

## Figures and Tables

**Table 1:** Demographic variables and length of rehab stay by vitamin D status.

Demographic	Total	WNL	Insufficient	Deficient	Sig.
Age (years) (mean ± sd)	65 (16.4)/19–90	69 (14.96)	62 (17.04)	62 (12.64)	0.015
Sex (% male)	66%	62%	71%	67%	0.517
Education (years) (mean ± sd)	14 (3.1)/5–23	14 (2.92)	14.3 (3.21)	13.7 (3.38)	0.339
Length of rehab stay (days)	19 (11.9)/1–104	17.1 (7.57)	18.9 (9.77)	22.2 (16.84)	0.018

**Table 2:** Group distribution, DVT occurrence, and supplementation in individuals with WNL and low vitamin D levels receiving acute inpatient rehabilitation for moderate–severe TBI.

Individuals with TBI *n* = 190			
WNL vitamin D level ≥ 30 ng/mL *n* = 73		Low vitamin D level < 30 ng/mL *n* = 117	
No DVT *n* = 67	DVT *n* = 6	No DVT *n* = 93	DVT *n* = 24

## Data Availability

The data presented in this study are available on request from the corresponding author. The data are not publicly available due to subject confidentiality.
